# *Chromobacterium violaceum*: A Rare Cause of Urinary Tract Infection

**DOI:** 10.1155/2021/5840899

**Published:** 2021-10-12

**Authors:** Ujjwal Laghu, Manami Yanagawa, Konosuke Morimoto, Bhim Gopal Dhoubhadel

**Affiliations:** ^1^Grande International Hospital, Kathmandu, Nepal; ^2^School of Tropical Medicine and Global Health, Nagasaki University, Nagasaki, Japan; ^3^Department of Respiratory Infections, Institute of Tropical Medicine, Nagasaki University, Nagasaki, Japan

## Abstract

A 41-year-old man with a neurogenic bladder due to spinal cord injury (SCI) attended the outpatient department with chief complaints of fever, pain in the lower abdomen, and persistent hematuria for 10 days. From the urine culture and the microbiological and biochemical tests, the causative organism was identified as *Chromobacterium violaceum*. The isolate was resistant to cephalosporins, while it was sensitive to ofloxacin, gentamicin, and imipenem. Clinicians should be aware of this rare cause of urinary tract infection (UTI), the choice of antibiotic, length of treatment, and necessity of prompt treatment in SCI patients.

## 1. Introduction

*Chromobacterium violaceum* is a Gram-negative betaproteobacterium, facultative anerobic, and oxidase-positive bacterium, motile by means of a single flagellum. It was first identified in 1881, and its pathogenic role was explained by Woolley in 1905 [1]. Its habitat is in soil and water in tropical and subtropical regions. The first human infection was reported in Malaysia in 1927 [2]. Only few cases of the infection have been reported since then [3]. The bacillus generally comes in contact with human body through ingestion of contaminated water or minor trauma. It is generally taken as an opportunistic pathogen, and it can cause fatal sepsis in immunocompromised individuals [4]. A median duration of clinical course among those who died is 7 days with the range of 1 to 97 days [5]. It is found to be highly resistant to penicillin and cephalosporins while sensitive to fluoroquinolones, chloramphenicol, trimethoprim/sulfamethoxazole, and carbapenem [5].

We present a case with a neurogenic bladder due to spinal cord injury who developed urinary tract infection caused by *Chromobacterium violaceum*. The neurogenic bladder is caused by both central and peripheral nervous system dysfunction including trauma-related spinal cord injury, cerebral palsy, multiple sclerosis, Parkinson's disease, and spina bifida. The patients with the neurogenic bladder have a higher risk of having recurrent urinary tract infection owing to urinary stasis, high bladder pressures, vesicoureteral reflux, bladder stone, and indwelling catheter use [6].

## 2. Case Presentation

A 41-year-old male attended the outpatient department with a chief complaint of fever, pain in the lower abdomen, and gross hematuria for 10 days. The patient had spinal cord injury (SCI) after falling from a tree of approximately 12 ft height 16 years back. Since then, he had difficulty in movement of both lower limbs. Palpation of the spine was tender on T7 downwards. The knee and ankle reflexes were absent in both sides. He had a neurogenic bladder, and an indwelling catheter was *in situ*. His past medical history showed that he had cholecystostomy 9 years ago.

Laboratory findings showed a hemoglobin level of 10.7 g/dL and a white blood cell count of 12,000/mm^3^. The differential count showed neutrophil of 57%, lymphocyte of 42%, eosinophil of 1%, and monocyte of 0%. Serum sodium and potassium levels were 136.0 mEq/L and 3.6 mEq/L, respectively. The serum blood sugar level was 66.6 mg/dL, and the urea level was 25.2 mg/dL. Blood culture was negative. The urine nitrite test was positive.

**Figure 1:**
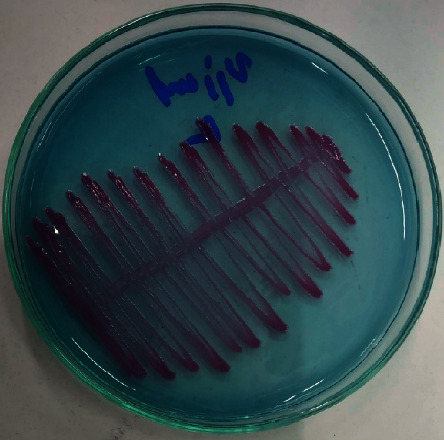
*Chromobacterium violaceum* in Cysteine Lactose Electrolyte Deficient agar.

Midstream urine was collected for urine culture aseptically. Pure colonies of the organism (>10^5^ CFU/mL) were grown in the Cysteine Lactose Electrolyte Deficient (CLED) agar, as shown in Figure 1. The colonies were 2 to 3 mm in diameter with nondiffusible violet color after an overnight incubation at 37°C. Facultative anerobic, catalase-positive, oxidase-positive, and Gram-negative bacteria were found and identified as *C. violaceum*. Urine culture was repeated next day, and *C. violaceum* was isolated and confirmed again.

The antibiotics susceptibility test was performed by the Kirby–Bauer disc-diffusion method, and the results are presented in Table 1. Among commonly used antibiotics in the hospital, the organism was sensitive to ofloxacin and gentamicin and resistant to cotrimoxazole, cefixime, and cephalexin.

The patient was given oral ofloxacin at a dose of 400 mg, two times a day, for 10 days. Among the sensitive antibiotics, ofloxacin could be given orally, and it was cheaper; therefore, it was selected. Ofloxacin is a fluoroquinolone; it is a bactericidal agent that inhibits the bacterial DNA gyrase and stops the DNA replication. It is a commonly used antibiotic in urinary tract infection. The patient's hematuria decreased gradually, and it completely subsided after the completion of treatment. Repeat urine culture was done at the end of the treatment, and it was negative.

## 3. Discussion

Most commonly identified organisms among patients with urinary tract infection in general population are *Escherichia coli*, *Klebsiella*, and *Proteus* species. In patients with a neurogenic bladder, aside from these organisms, nosocomial bacteria, including *Pseudomonas aeruginosa*, *Acinetobacter* spp., *Enterococcus* spp., and *Staphylococcus* spp. have a high incidence [7]. In this patient, *C. violaceum* was the only organism confirmed from the laboratory result.

*C. violaceum* infection is unusual in healthy people [8]. Human infection generally occurs in tropical and subtropical areas in the summer season. It is considered as the organism of low virulence in healthy people. But, under the immunocompromised condition, this organism can prove to be fatal. The organism gets access to the body by minor trauma from contaminated water or soil. Other possible routes of infections include oral route by consumption of contaminated food or water, surgery, road traffic accidents, urinary catheterization, and nosocomial infections [1, 9]. Our patient had SCI with the neurogenic bladder with an indwelling catheter *in situ*. He presented with symptoms of UTI, and urine culture showed *C. violaceum.* Although it is a rare cause of UTI, treating doctors need to be aware of it, particularly when treating empirically, as this pathogen is resistant to many commonly used antibiotics.

People with SCI show increased risk of UTI [10]. In the animal model, it was indicated that SCI affects the immune response and makes the host susceptible to infections [11]. In another animal model using *E. coli*, it was implied that patients with the neurogenic bladder caused by SCI might require less bacterial load to develop an infection. The same model also showed that once infection is established, it will be prolonged in the SCI individual due to delayed clearance of bacteria [6]. Although very little is known about the mechanism of how UTI is prone to occur and causes severe consequences in the neurogenic bladder, these findings might explain a probable increased susceptibility of the neurogenic bladder to *C. violaceum* infection.

Rapid progression to sepsis and multiorgan dysfunction along with high mortality rate are the main features of *Chromobacterium* infection [12]. Since only few cases of urinary tract infection caused by *C. violaceum* has been reported till now, the availability of its antibiogram is limited. Hence, selection of antibiotics is crucial and should be based on the sensitivity pattern to prevent from the fatal outcome. Besides, we need to keep in mind that the incidence of multidrug-resistant bacterial infection is higher among patients with SCI than in healthy population due to repeated antibiotics exposure [6]. It is reported that nearly half of the causative agents identified among UTI patients with the neurogenic bladder were resistant to fluoroquinolone [7]. Therefore, urine culture with antimicrobial susceptibility testing is essential before treating UTI in SCI patients.

## 4. Conclusions

Urinary tract infection caused by *C. violaceum* is uncommon. Patients with SCI are more prone to infection than normal population. Humans get infection by invasion through minor trauma or inoculation of contaminated soil and water. *C. violaceum* can cause UTI and sepsis that may lead to fatal multiorgan failure. As *C. violaceum* can be resistant to commonly used antibiotics, culture and antimicrobial susceptibility testing are necessary before treatment. Hence, clinicians should be aware of the effective antibiotics, duration of treatment, and prompt treatment of UTI in SCI patients.

## Figures and Tables

**Table 1:** Antimicrobial susceptibility test result.

Antimicrobial agent	Susceptibility result^#^
Piperacillin-tazobactam	Sensitive
Cephalexin	Resistant
Cefazolin	Resistant
Ceftriaxone	Resistant
Cefixime	Resistant
Meropenem	Sensitive
Imipenem	Sensitive
Aztreonam	Sensitive
Ofloxacin	Sensitive
Norfloxacin	Resistant
Cotrimoxazole	Resistant
Gentamicin	Sensitive
Nitrofurantoin	Resistant

^#^Sensitive antibiotics are generally given for 7 to 14 days according to the severity of urinary tract infection.

## Data Availability

No data were used to support this study.
